# Health and Holiness
*The Healing Art the Right Hand of the Church; or, Practical Medicine an Essential Element in the Christian System. By Therapeutes (Edinburgh : Sutherland and Knox. London : Simpkin, Marshall, and Co.) 1859.


**Published:** 1894-08-25

**Authors:** 


### Health and Holiness.*

In the early days of the world the priest and the physician were one man. The oldest books of India are partly theological, partly therapeutic, and in the Levitical law a large proportion of the instructions deal with the bodies rather than the souls of the Israelites. It is probable that even observances regarded ultimately as purely ceremonial were begun for more practical reasons. Circumcision itself, the national mark of the Hebrew amid his heathen environment, may well have been in its initiation a measure for the prevention or relief of disease. Unfortunately, the two professions have long been divorced, and what Sir Thomas Browne calls "the common scandal of my profession" crystalised in the proverb *ubi tres medici, due athei*, still holds good.

The priest is primarily to blame for this. The first pious worker naturally and truly takes his observations and the happy result of his experiments as a revelation from the Deity. His successor, less pious and less observant, accepts the revelation, now a tradition, handed down to him, as complete and ultimate, not realising that "God reveals Himself in many ways," and mocks or burns, according to His temper and His power, that other observer and experimenter, unclerical, who uses a different process to cure the same disease. The first priest accompanied his treatment with prayers; the second keeps the prayers and omits the treatment; while the unfrocked physician, called witch or herbalist, and liable at any moment to the punishment of sorcery, sees his treatment succeed without the prayers, and becomes the atheist of the proverb. O foolish priest, does He who made the whole world with man for only part, if yet the noblest part of His creation, care for nought in the world but the utterances, at best how feeble, of man's heart and imagination! O supercilious physician, who taught thee to observe symptoms and apply remedies? Who planted in these remedies the virtue they possess?

It is only in these latter days that any re union of the two professions has been attempted. The book which we have taken as our text is a serious attempt, founded on no little study of the past to bring priest and physician into a proper relation. It was published in 1859, and won the commendation of even that least easily satisfied of philosophers, Thomas Carlyle. We are privileged to subjoin a letter, hitherto unpublished, from Carlyle to the author:—

Chelsea, February 25th, 1859.

Dear Sir,—I have received your book which you were kind enough to send me, and I beg to return you thanks for the same. It is a book (unlike many that come to me here) of a serious nature, the fruit of long study, meditation, inquiry, and evidently of perfect conviction on your part.

I believe, and have long believed, the essential idea it sets forth to be not only true, but of the very highest importance to mankind, namely, that the physician must first of all be a priest (that is to say, a man of pious nobleness, devoted to the service of the Highest, and prepared to endure and endeavour for that same, taking no counsel of flesh and blood, as the theory of priests is)—first of all, a real priest, and then that the whole world should take supreme counsel of him, as it does of its real or imaginary priests or pontiffs this long while back, and follow said counsel as the actual will of God—which it would be were the physician what I say.

It is curious to remark that Heilig in our old Teutonic speech is both holy and also healthy; that the words holy and healthy, as our antique fathers understood them, are one and the same. A thousand times has that etymology risen sorrowfully upon me in looking at the present distracted position of affairs, which is horrible to think of if we look earnestly into it, and which cannot well be spoken of at all. We, sure enough, have completely contrived to divorce holiness (as we call it) from health, and have been reaping the fruits very plentifully during these fifteen hundred years.

The notion of bringing our present distracted anomaly of a physician into union with our ditto ditto of a priest, and making them identical is, of course, extremely chimerical; nor can one easily say what ought to be the first step towards bringing each of them back from his anomalous imaginary condition and nearer to veracity and the possibility of coalescing. But I am very glad to see the idea started in any form, under any vesture, and heartily wish you success in bringing it home to men's mind.—I remain, yours truly,

T. Carlyle.

In our own country and circumstances it is hardly possible, nor indeed desirable, to combine both offices in the one person, but while perhaps the priest will be wise to meddle as little as possible with the functions of the doctor, the doctor cannot avoid to some extent fulfilling those of priest. He is the confessor of all his patients, and knows in what proportion mind and body cause their ailments. He knows, too, that disease is the result of sin, some outrage of the natural law direct or vicarious, and can advise to temperance and cleanliness of life at the moment when such advice appeals most strongly to the conscience and the brain. Moreover, he will avoid extremes more shrewdly than the mere theologian inclines to do. He will advise temperance but not asceticism, chastity but not enforced celibacy, self-denial for health or duty's sake but not self-surrender to the caprices of selfish individuals.

But abroad, in that vast and growing heathen empire which is ours, the missionary and the doctor may well be one. A Livingstone and a Duff leave their impress on India and Africa, because they show that they have the power as well as the will to help the people they dwell among. There is no stronger incitement to conversion than St. Peter's invocation, "In the name of Jesus of Nazareth, rise up and walk," But let the missionary be sure that he is first known and trusted as the healer of diseases, not primarily suspected to be the insidious propagator of an alien faith. We cannot blame the conscientious Mahommedan or Hindoo, even the savage but sincere worshipper of Mumbo Jumbo, who lets his dear ones suffer and die rather than have them imperil their souls (as he thinks) by accepting the strangers' creed. Let the missionary prove himself a true physician, and his works and the spirit that leads him to spend and be spent for these his brethren in foreign lands will speak more strongly than any words for the divinity of the Master whom he serves.

*The Healing Art the Right Hand of the Church; or, Practical Medicine an Essential Element in the Christian System. By Therapeutes (Edinburgh: Sutherland and Knox. London: Simpkin, Marshall, and Co.) 1859.

